# Sugar for Cleaning Hands

**Published:** 1914-05

**Authors:** 


**Sugar for Cleaning Hands,** instead of corn meal is advocated by Douglas H. Stuart, Med. Council, Jan., 1914.

